# The Effect of Activity Tracking Apps on Physical Activity and Glycemic Control in People with Prediabetes Compared to Normoglycemic Individuals: A Pilot Study

**DOI:** 10.3390/nu17010135

**Published:** 2024-12-31

**Authors:** Aikaterini Kalampoki, Evangelia E. Ntzani, Alexandros-Georgios I. Asimakopoulos, Evangelos Liberopoulos, Nikolaos Tentolouris, Georgia Anastasiou, Petros-Spyridonas Adamidis, Kalliopi Kotsa, Evangelos C. Rizos

**Affiliations:** 1 School of Health Sciences, University of Ioannina, St. Niarchou Av, 45500 Ioannina, Greece; katerinakal98@yahoo.com; 2 Department of Hygiene and Epidemiology, University of Ioannina School of Medicine, 45500 Ioannina, Greece; entzani@uoi.gr; 3 Center for Evidence-Based Medicine, Department of Health Services, Policy and Practice, School of Public Health, Brown University, Providence, RI 02903, USA; 4 Department of Primary Education, University of Ioannina, 45500 Ioannina, Greece; a.asimakopoulos@uoi.gr; 5 School of Medicine, National & Kapodistrian University of Athens, 11527 Athens, Greece; elibero@med.uoa.gr (E.L.); ntentol@med.uoa.gr (N.T.); 6 Department of Internal Medicine, Faculty of Medicine, School of Health Sciences, University of Ioannina, 45500 Ioannina, Greece; anastgeorgia.94@gmail.com (G.A.); padam7@yahoo.gr (P.-S.A.); 7 School of Medicine, Aristotle University of Thessaloniki, 54124 Thessaloniki, Greece; kkalli@auth.gr

**Keywords:** prediabetes, physical activity, activity tracking apps, Google Fit

## Abstract

Introduction—Aim: Adopting a lifestyle that incorporates regular physical activity confers substantial benefits to both physical and mental health and is recommended for prediabetic individuals. The aim of this study is to investigate the impact of activity tracking apps on increasing physical activity and its effect on glycemic control in people with prediabetes. Materials and Methods: This pilot study included 37 participants, 18 in the prediabetic group and 19 in the normoglycemic group matched for age and gender (mean age 53 years, 40% males). Participants used the Google Fit app for 3 months. The number of daily steps was recorded via the app, and blood and urine tests as well as body fat measurements were conducted before and following 3 months of app use. The co-primary outcome was the change in steps, and the change in HbA1c in both groups. Secondary outcomes were the change in fasting plasma glucose (FPG) (main secondary outcome), as well as lipid parameters, body mass index, visceral fat, and kidney function indices among the two groups. Results: Both groups increased the daily step count following the app intervention, without any statistically significant difference when we compared the steps change between the two groups. We found a statistically significant difference between HbA1c levels in favor of the prediabetic compared to the normoglycemic group [mean difference (MD) 0.16, 95%CI 0.04, 0.28, *p*-value 0.01)], following the 3-month intervention. Additionally, there was a statistically significant difference between FPG levels in favor of the prediabetic compared to the normoglycemic group (MD 9.06, 95%CI 4.16, 13.96, *p*-value 0.001). Conclusions: The present study suggests that the use of activity tracking apps, combined with tailored activity goals and monthly supportive phone calls, may contribute to improved glycemic control among individuals with prediabetes.

## 1. Introduction

Physical activity plays a crucial role in reducing cardiovascular (CV) risk as well as all-cause mortality [1]. Globally, one in four adults does not meet the physical activity recommendations set by the World Health Organization [2]. Insufficient physical activity is a significant risk factor for chronic diseases, mental health issues, and, consequently, a diminished quality of life [3]. Cardiovascular disease (CVD) continues to be the leading cause of disability and mortality worldwide, whereas the incidence of obesity, type II diabetes (T2D), hypertension, colorectal cancer, breast cancer, depression, and osteoporosis is increasing [4,5,6]. Numerous epidemiological studies have concluded that physical activity provides significant protective benefits for patients facing these health issues [4,5,6,7]. In particular, adopting a modified lifestyle that includes 150 min of moderate-intensity exercise per week—primarily through walking—can reduce the risk of developing T2D by approximately 58% [8].

Wearable activity trackers and apps are part of e-coaching lifestyle applications and have the potential to successfully intervene in changing people’s behavior, including their levels of physical activity [9]. These tools provide individuals with the ability to monitor their activity levels and performance over time, and they also offer the option to track food intake [10]. Research has demonstrated that activity tracking apps have shown effectiveness in increasing physical activity levels and improving health markers, such as body mass index (BMI), by promoting self-monitoring and goal setting [11,12]. These apps frequently include features such as social sharing, gamified elements, and personalized feedback, enhancing user engagement and motivation by fostering social support and healthy competition [13]. While short-term adherence is generally high, long-term impact may be limited as initial motivation declines, underscoring the importance of adaptive, personalized features to maintain sustained engagement [14].

Prediabetes is defined as glucose levels higher than normal but lower than diabetes threshold with diagnostic criteria including glycated hemoglobin (HbA1c) levels between 5.7% and 6.4%, fasting plasma glucose (FPG) levels between 100–125 mg/dL, or serum glucose levels between 140–199 mg/dL at 2 h following an oral glucose tolerance test (OGTT) with 75g of glucose, according to American Diabetes Association (ADA) definition [15]. Prediabetes is closely associated with the development of T2D [15]. Approximately 35% of adults in the U.S. live with prediabetes. Regular exercise helps prevent the progression of prediabetes to T2D [8]. According to the European Society of Cardiology (ESC) guidelines, a physical activity increase is recommended for all patients with T2D, with or without CVD. They also suggest that increased physical activity is promoted using activity trackers [16].

So far, limited reports have assessed the effect of activity tracking apps in prediabetic individuals. The first approach on e-coaching lifestyle modification is the use of simple apps, which usually incorporate data on diet and physical activity. This intervention showed neutral effect or benefit on weight, lipid, and HbA1c levels [16,17,18]. The second approach, which uses more sophisticated tools based on AI, showed the same pattern of neutral effect or favorable changes in weight and HbA1c levels [19,20,21]. On the other hand, physical activity and its effectiveness in prediabetes have been studied thoroughly, showing that combining moderate-intensity aerobic exercise with low-to-moderate-load resistance training yields significant improvements in HbA1c, BMI, total, and LDL cholesterol [17], whereas intensive lifestyle interventions reduced by 22% the risk for progression to T2D [18].

Although there is substantial research interest, the number of studies addressing the effect of e-health tools in prediabetic subjects remains significantly limited. This study aims to investigate the impact of activity tracking apps on physical activity and metabolic parameters among individuals with prediabetes compared to normoglycemic individuals.

## 2. Materials and Methods

### 2.1. Participants

The sample for this pilot study consisted of prediabetic and healthy (normoglycemic) individuals matched for age and sex. The study sample was recruited from the outpatient lipid, diabetes, and internal medicine clinic of the university hospital of Ioannina, Greece. Inclusion criteria were based on the glycemic profile of the participants and we use as threshold for prediabetes the American Diabetes Association (ADA) criteria [15]: normoglycemic (FPG < 100 mg/dL, serum glucose < 140 mg/dL at 2 h following OGTT with 75 g of glucose, HbA1c < 5.7% [19]) or prediabetic (FPG 100–125 mg/dL, serum glucose 140–199 mg/dL at 2 h following OGTT with 75 g of glucose, HbA1c 5.7–6.4% [15]). Voluntary participation in the study, ownership of a smartphone, and the ability to use it was required. Patients receiving anti-diabetic medications or suffering from any type of chronic disease including malignancy were excluded. Finally, the participants should not use an app or any type of wearable activity tracker during the last 3 months. Participants were informed about the study and signed the informed consent form.

### 2.2. Intervention—Data Collection

Participants completed a demographic questionnaire (Figure S1) and underwent laboratory tests of blood and urine, which included FPG, HbA1C, cholesterol levels, and urine albumin to creatinine ratio (ACR). Additionally, body fat analyses were conducted using a Tanita body composition monitor (MC580S, Tanita Corporation, Tokyo, Japan) to record body mass index (BMI) and visceral fat. The Google Fit app was installed on the participants’ smartphones, and the first week’s step count results were considered as the baseline measurements. The Google Fit app version 1.85 (Google Inc., Mountain View, CA 94043, USA) allows users to manage their fitness data free of charge [20]. It employs advanced sensors such as an accelerometer, gyroscope, and GPS system to detect changes in position, different types of movements, and data, as well as specific periods of activity [20]. Google Fit application is a valid tool to track physical activity and has a stronger correlation with measurements taken by the examiner compared to the data gathered from the GT3X ActiGraph accelerometer [20]. The daily step count was recorded via the Google Fit smartphone application and adapted goals were established in accordance with the latest ESC guidelines [16]. According to the 2023 ESC guidelines for managing CVD in T2D, an increase in any form of physical activity is recommended for all subjects with T2D, whether or not they have established CVD [16]. It is advised that all individuals aim for more than 150 min of moderate-intensity physical activity or more than 75 min of vigorous exercise per week [16]. Every month there was a telephone call with each participant inquiring about the use of the app, explaining any issues and encouraging them keep using it to promote physical activity. All laboratory tests and body fat measurements were repeated after 3 months of the initial visit, allowing enough time for parameters such as HbA1c to change [21]. Any change in underlying medications was not allowed during the study. No dietary change instructions were given to participants, and they did not record their food intake.

Blood tests (FPG, insulin, HbA1c, creatinine, total cholesterol (TCHOL), triglycerides (TRG), high density lipoprotein cholesterol (HDL-C), low density lipoprotein cholesterol (LDL-C), apolipoprotein (APO) A1, APO B, lipoprotein a (Lpa) and vitamin D), urine tests (albumin-to-creatinine ratio (ACR)), body fat measurements (BMI and visceral fat) were measured, and step count measurements (Google Fit app) were recorded from the total study sample at baseline (t0) and during the final visit 3 months later (t1).

### 2.3. Hypothesis—Study Outcomes

It is known that approximately 1/3 of prediabetics will remain in the same category in the next 5 years, 1/3 will proceed to diabetes (5–10% annual conversion rate, which varies by population characteristics and the definition of prediabetes), and 1/3 could return to normoglycemia, provided that lifestyle modification is successful [22]. The rational of this study is to assess the effect of the app in prediabetics compared to normoglycemics, provided that the app would have the same effect on increasing physical activity in the 2 groups of interest. If indeed there is a differential effect on glycemic control from the app intervention between the 2 groups, this finding could serve as a proof of concept for using such interventions to delay the progression from prediabetes to diabetes or even regression to normoglycemia (provided that lifestyle has dramatically changed and values of HbA1c and glucose are closer to the lower cut-off of prediabetes range).

The co-primary outcome was the change in steps, as well as the change in HbA1c in both groups.

Secondary outcomes were the change in FPG (main secondary outcome), as well as lipid parameters, BMI, visceral fat, and kidney function indices in the 2 groups.

### 2.4. Statistical Analysis

A variable representing the difference between the second and the first measurement for each indicator was created and then the difference between the final and baseline measurements for each parameter was calculated. The normality of the difference variables was assessed using the Shapiro–Wilk test, and either *t*-test (for normally distributed populations) or Mann–Whitney U test was applied to examine statistically significant differences between prediabetic and normoglycemic groups for both primary and secondary outcomes. We conducted a linear regression model as a sensitivity analysis for the association between the post-test scores of each indicator and the intervention adjusting for the pre-test scores. Power calculations were also performed for co-primary outcomes. Statistical analyses were conducted using STATA 17 (StataCorp LP, College Station, TX, USA) software [23].

## 3. Results

We included 37 participants (range 40–60 years), 18 in the prediabetic group (mean age 53.8 years), and 19 in the normoglycemic group (mean age 51.9 years). Both groups had a similar gender ratio (prediabetics: 44% male; normoglycemics: 42% male).

Among prediabetic individuals, 72% were receiving some form of medication such as statins (72%), antihypertensives (28%), or were using supplements of vitamin B12, D3, calcium, or thyroxine (5%). The demographic questionnaire completed by participants before engaging in the intervention showed that 28% of those with prediabetes reported not following an active lifestyle or exercising regularly during the week, 50% reported exercising twice a week, 17% three times, and 5% four times a week. Of those who exercised, 50% spent 30 min exercising during each session, while the remaining 50% spent 60 min exercising. When asked about the type of exercise they engage in, the majority mentioned walking (65%), followed by Pilates, gym workouts, and running. Finally, 44% of the prediabetics had used some form of activity tracking app in the past. As regards the normoglycemic group, 32% of them were receiving statins. The demographic questionnaire completed by normoglycemic participants showed that 37% of those reported not following an active lifestyle and not exercising at all during a week, 5% exercised once a week, 32% twice, 21% three times, and 5% four times a week. Among those who exercised, 33% spent 30 min during each session, while the remaining 67% spent 60 min exercising. When asked about the type of exercise they engage in, the majority mentioned walking (50%), followed by running, gym workouts, Pilates, and dancing. Ultimately, 37% of the normoglycemic subjects had used some form of activity tracking app in the past (Table 1).

Baseline and final values of the lab tests, as well as BMI, visceral fat, and step count measurements are presented in Table 2. The two groups did not significantly differ when baseline values were compared, except for HbA1c and FPG, which were significantly lower in the normoglycemic group, as expected (Table S1). Even though age and sex were the main factors for matching during sampling, BMI, which is associated with glycemic control, did not significantly differ.

The power analysis yielded a 6% power for change score of HbA1c with control mean value of −0.144 and a 0.1% difference between the control group and experimental group means. The power for change score of steps with control mean 407.33 and difference of 320 steps was reached to 99%. There was a statistically significant change between pre-time and post-time score of HbA1c (*p*-value = 0.019) and non-significant result of steps (*p*-value = 0.313) in the prediabetic group. A statistically significant difference of the HbA1c change (the other co-primary outcome) in favor of the prediabetic compared to the normoglycemic individuals [mean difference (MD) 0.16, 95%CI 0.04, 0.28, *p*-value 0.01)] was found, following the 3-month intervention. Moreover, there was a statistically significant difference between FPG levels in favor of the prediabetic compared to the normoglycemic group (MD 9.06, 95%CI 4.16, 13.96, *p*-value 0.001). The comparison of the difference between baseline and final measurements for the other parameters did not show any significant results (Table 3). Concerning sensitivity analysis, the statistical significance of the results remains the same as the main analysis, except for the association between the intervention and the final measures of the FPG, which is indicated as non-significant (Table S2).

As was stated by the monthly supportive phone call, participants engaged with the Google Fit app daily over the three-month period. This regular use enabled them to easily monitor their progress and adjust their activity levels with the recommended goals. With features like daily reminders and progress tracking, the app likely helped keep participants motivated, supporting them steadily to pursue their weekly activity goal.

## 4. Discussion

We found that the Google Fit app was associated with notable improvements in glycemic control among individuals with prediabetes over a three-month period, even though the increase in physical activity levels was similar between the prediabetic and normoglycemic groups. Although the activity tracking app effectively encouraged people to become more active, its impact is more pronounced in those with higher baseline risk for metabolic dysfunction, such as individuals with prediabetes [24,25].

Physical activity, a key lifestyle intervention, has been shown to positively impact the prevention and management of various conditions including hypertension, dyslipidemia, obesity, diabetes, CVD, and mental or psychological health [26]. In the United States, inadequate physical activity—defined as not meeting established guidelines for aerobic physical activity—is associated with approximately USD 117 billion in annual healthcare costs and around 10% of premature deaths [27,28,29]. Globally, 374 million individuals are at an increased risk of developing T2D, a number expected to rise to 700 million by 2045 due to factors such as urbanization and population aging [30]. People with prediabetes are at increased risk of developing T2D (15). Lifestyle changes aim to decrease weight and increase physical activity are the cornerstones of prediabetes management [15]. Even a small weight reduction of 5% can be associated with increased insulin sensitivity and improved glycemic control in prediabetic subjects, as well as reduced dependence on diabetic medications in T2D patients [31,32]. Adopting a lifestyle that includes 150 min of moderate-intensity physical activity per week, primarily through walking, can reduce the risk of developing T2D by approximately 58% [8]. New health-promoting technologies through electronic devices can be beneficial in helping individuals to achieve this goal [16]. We found an improved glycemic control expressed by reductions in HbA1c and FPG in prediabetic subjects indicating that, while both groups increased their step count, the prediabetic group have gained substantial glycemic benefits. The 0.16% difference in HbA1c between the two groups is clinically relevant, since a 1% reduction in HbA1c levels is associated with a 21% decrease in mortality, a 14% reduction in the risk of myocardial infarction, and a 37% lower likelihood of developing microvascular complications [33]. In relation to our study, e-coaching lifestyle modification has used either simple apps or more sophisticated tools based on AI. In the first case, e-coaching by using simple apps resulted in weight loss and improved glycemic control in 171 individuals with diabetes or prediabetes when the Nutritionist Buddy Diabetes app was used for >6 months and >90% engagement. Those participants who actively used ≥5 app features experienced the greatest weight loss of 11% and significant reductions in HbA1c levels [34]. In a similar study, 30 prediabetics monitored their physical activity using Fitbit activity trackers. After 6 months, there was no significant change in the overall physical activity, although significant correlations were found between changes in lipid levels or BMI and fluctuation in physical activity levels [35]. Moreover, when the variability of physical activity was assessed using Fitbit Inspire devices in 30 prediabetic individuals, both HbA1c and lipid levels were favorably changed [36]. In the second case, e-coaching lifestyle modification by using more sophisticated tools (AI algorithms) managed to lower their weight and BMI, but not HbA1c [37]. In a study where continuous glucose monitoring (CGM) was applied in a mixed population of 2217 normoglycemic, prediabetic, or diabetic subjects and analyzed the data on glycemic status, food intake, macronutrients components, and physical activity through AI tools, the results were encouraging regarding the glycemic control and weight loss [38]. When a fully automated, personalized mHealth platform (Sweetch) was tested in 55 prediabetic individuals, the physical activity was increased, whereas HbA1c was decreased [39].

In the present study, the prediabetic group experienced significant reductions in both HbA1c and FPG compared to the normoglycemic group following the intervention with an activity tracking app, which managed to increase the daily step count. This study suggests that activity tracking apps could be a valuable adjunct tool for glycemic control in prediabetic individuals. These findings underscore the potential of activity-tracking apps to support meaningful glycemic improvements in individuals with prediabetes, possibly by encouraging sustained increases in physical activity and fostering a greater commitment to lifestyle modification [40,41]. Thus, the progression of prediabetes to diabetes might be delayed. No significant differences were observed in other metabolic or cardiovascular markers, including BMI, visceral fat, lipid profiles, and kidney function indices, between the two groups. These findings may reflect the short duration of the intervention, as changes in body composition and lipid metabolism often require longer periods to become apparent [24].

Our study stands out by the simplicity of the approach, which uses one of the commonest—free of charge—app that targets physical activity, compared to the previous reports that used sophisticated tools (some requiring subscription), or included mixed populations, or demanded additional data based on dietary habits. With the rising rates of prediabetes and the risk of progression to T2D, accessible and scalable tools such as activity tracking apps could be crucial in public health. Healthcare providers may consider recommending such apps as part of the holistic approach for lifestyle modification, as they offer an efficient and easy-to-use way to help individuals set physical activity goals and monitor their progress [40,41]. While activity trackers can motivate physical activity, several challenges limit their effectiveness for long-term health behavior change. A key challenge is user engagement, as many individuals stop using the trackers after a few months due to waning motivation or the novelty wearing off [42]. The effectiveness of activity trackers in driving behavior change is variable, depending on factors such as initial activity levels, motivation, and health literacy [43]. This variability may limit their effectiveness in certain populations. While activity trackers record physical activity, they fail to capture other essential behaviors related to health, such as diet and stress [44]. Privacy concerns related to data collection and the potential for breaches also pose ethical dilemmas [45]. Finally, e-illiteracy is frequently underestimated, as many people commonly experience difficulties when navigated through the app features and the understanding of the app language, particularly among those with lower educational levels and socioeconomic status [46].

While our study demonstrates the potential of activity tracking apps in supporting glycemic control in prediabetes, certain limitations warrant consideration. Our population sample was derived through targeted sampling, which, in turn, raises the issue of randomization and objectivity in the sampling process. The three-month duration of the study, while sufficient to observe changes in glycemic measures, may have been too short to detect more gradual changes in other metabolic or CV markers such as BMI, visceral fat, lipid profiles, or kidney function indices. The differences between the two groups in baseline lipid levels, BMI, visceral fat, and renal function is attributed to the selection process, which was based on glycemic profile and was matched for age and sex. Importantly, these differences were non-statistically significant. Future research on larger sample sizes, longer follow-up, and more homogeneous populations is needed. However, the participants of the two groups were age- and gender- matched, and most of the evaluated parameters did not significantly differ during baseline measurements, except for those that were normally expected (FPG, HbA1c). The absence of blinding may have influenced participant behavior, as knowledge of being monitored might have affected their responses or actions. Finally, the dietary patterns were not assessed in this study, which could be a confounding factor in interpreting glycemic improvements. Dietary changes, commonly advised in managing prediabetes, could enhance the benefits seen from increased physical activity and the use of tracking apps. Future studies that incorporate dietary tracking along with activity monitoring could provide a more comprehensive view of lifestyle modification effects on glycemic control in prediabetes. Despite these limitations, the use of a single activity tracking application, the analysis of laboratory tests in a single laboratory, and the collection of anthropometric data through body fat measurements under the same conditions using the same body fat analyzer further strengthens the validity of our findings.

## 5. Conclusions

The present study suggests that the use of activity tracking apps, combined with tailored activity goals and monthly supportive phone calls, may contribute to improved glycemic control in prediabetic participants. Their use should be recommended as an adjunctive tool for lifestyle modification in both prediabetic and normoglycemic subjects. Future research focused on improved activity-enhancing interventions by incorporating additional innovations in this area could be of paramount importance towards an improved glycemic control among prediabetic individuals.

## Figures and Tables

**Table 1:** Baseline characteristics of the participants.

N all = 37	Prediabetics (N = 18)	Normoglycemics (N = 19)
**Age (mean years)**	53.8	51.9
**Females**	10 (56%)	11 (58%)
**Lipid-lowering therapy—statins**	13 (72%)	6 (32%)
**Antihypertensive therapy**	5 (28%)	-
**Number of days of exercise per week**		
0	5 (28%)	7 (37%)
1	-	1 (5%)
2	9 (50%)	6 (32%)
3	3 (17%)	4 (21%)
4	1 (5%)	1 (5%)
**Exercise duration per session (among those who exercise)**	N = 14	N = 12
30 min (%)	7 (50%)	4 (33%)
60 min (%)	7 (50%)	8 (67%)
**Type of exercise (among those who exercise)**	N = 14	N = 12
Walking	9 (65%)	6 (50%)
Running	1 (7%)	2 (17%)
Pilates	2 (14%)	1 (8%)
Gym workout	2 (14%)	2 (17%)
Dancing	-	1 (8%)
**Previous use of activity tracking app**		
Yes	8 (44%)	7 (37%)
No	10 (56%)	12 (63%)

**Table 2:** Baseline and final measurements [absolute values, mean and standard deviation (SD), or median and 25–75 interquartile range (IQR)].

	Normoglycemics [Mean (SD) or Median (25–75 Percentile)]		Prediabetics [Mean (SD) or Median (25–75 Percentile)]	
	Baseline (t0)	Final (t1: 3 Months)	Baseline (t0)	Final (t1: 3 Months)
**Steps/day**	6292 (3245.8)	7114 (2763.7)	6360 (2782.4)	6767 (2818.5)
**BMI (kg/m^2^)**	31.1 (27.20; 33.40)	31.1 (27.10; 33.00)	28.3 (25.05; 30.95)	28.0 (24.90; 31.63)
**Visceral fat (kg)**	10.42 (3.92)	10.16 (4.02)	9.83 (3.55)	9.78 (3.73)
**FPG (mg/dL)**	86 (10.2)	86 (9.3)	109 (6.5)	99 (10.4)
**Insulin (mUI/mL)**	6.50 (3.50; 8.90)	6.50 (3.30; 9.80)	10.05 (6.60; 11.90)	8.45 (4.90; 10.48)
**HbA1c (%)**	5.3 (0.32)	5.3 (0.31)	5.9 (0.24)	5.8 (0.36)
**Cre (mg/dL)**	0.86 (0.14)	0.89 (0.13)	0.89 (0.14)	0.92 (0.12)
**TCHOL (mg/dL)**	204 (154; 223)	198 (163; 208)	156 (143; 230)	153 (141; 182)
**TRG (mg/dL)**	90 (69; 114)	79 (65; 100)	94 (74; 131)	92 (80; 120)
**HDL-C (mg/dL)**	58 (16.1)	59 (14.5)	55 (15.0)	53 (14.1)
**LDL-C (mg/dL)**	122 (90; 142)	113 (95; 136)	89 (74; 135)	81 (69; 108)
**APOA1 (mg/dL)**	157 (24.7)	158 (20.4)	152 (30.3)	153 (29.5)
**APOB (mg/dL)**	80 (76; 93)	80 (78; 90)	73 (64; 89)	74 (62; 87)
**Lpa (mg/dL)**	6.1 (2.7; 28.7)	6.2 (2.7; 26.9)	9.3 (3.9; 40.1)	11.4 (3.8; 45.5)
**ACR (mg/g)**	6.2 (2.32)	6.7 (2.86)	10.4 (12.04)	9.9 (10.37)

ACR: albumin/creatinine ratio, APOA1: apolipoprotein A1, APOB: apolipoprotein B, BMI: body mass index, Cre: creatinine, FPG: fasting plasma glucose, HbA1c: glycated hemoglobin, HDL-C: high-density lipoprotein cholesterol, LDL-C: low-density lipoprotein cholesterol, Lpa: lipoprotein (a), TCHOL: total cholesterol, TRG: triglycerides.

**Table 3:** Comparison between the changes of baseline and final measurements between the two groups.

	Normoglycemics (Change t1–t0) [Mean (SD) or Median (25–75 Percentile)]	Prediabetics (Change t1–t0) [Mean (SD) or Median (25–75 Percentile)]	Difference (Mean, 95%CI) Between Changes (t1–t0) of Normoglycemics and Prediabetics	*p*-Value
**Steps/day**	822 (1532.0)	407 (1661.1)	414 (−614.80; 1442.80)	0.44
**BMI (kg/m^2^)**	0.00 (−0.30; 0.00)	−0.05 (−0.63; 0.60)	0.05 (−0.14; 0.23)	0.61
**Visceral fat (kg)**	−0.26 (0.45)	−0.06 (1.00)	−0.21 (−0.7; 0.28)	0.42
**FPG (mg/dL)**	−0.05 (7.78)	−9.11 (7.42)	9.06 (4.16; 13.96)	**0.001**
**Insulin (mUI/mL)**	−0.10 (−0.40; 0.10)	−0.15 (−3.87; 0.25)	−0.05 (−6.07; 5.97)	0.76
**HbA1c (%)**	0.02 (0.06)	−0.14 (0.24)	0.16 (0.04; 0.28)	**0.01**
**Cre (mg/dL)**	0.04 (0.07)	0.03 (0.06)	0.001 (−0.038; 0.040)	0.95
**TCHOL (mg/dL)**	−9.0 (−15.0; −1.0)	−5.5 (−46.8; 3.8)	−3.5 (−68.20; 61.20)	0.92
**TRG (mg/dL)**	−5.0 (−15.0; 3.0)	−11.5 (−30.3; 25.3)	6.5 (−270.45; 283.45)	0.96
**HDL-C (mg/dL)**	1.0 (6.38)	−1.9 (5.53)	2.89 (−1.32; 7.10)	0.15
**LDL-C (mg/dL)**	−5.0 (−12.0; 2.0)	−5.0 (−29.5; 2.0)	0.001 (−0.005; 0.007)	0.76
**APOA1 (mg/dL)**	1.58 (10.88)	0.33 (11.39)	1.25 (−5.92; 8.42)	0.74
**APOB (mg/dL)**	−0.80 (−3.20; 1.20)	−0.20 (−2.15; 3.27)	−0.6 (−3.01; 1.81)	0.63
**Lpa (mg/dL)**	0.00 (−0.10; 1.40)	−0.10 (−1.18; 1.97)	0.1 (−0.08; 0.28)	0.29
**ACR (mg/g)**	0.56 (2.57)	−0.44 (3.40)	0.99 (−0.93; 2.91)	0.32

ACR: albumin/creatinine ratio, APOA1: apolipoprotein A1, APOB: apolipoprotein B, BMI: body mass index, Cre: creatinine, FPG: fasting plasma glucose, HbA1c: glycated hemoglobin, HDL-C: high-density lipoprotein cholesterol, LDL-C: low-density lipoprotein cholesterol, Lpa: lipoprotein (a), TCHOL: total cholesterol, TRG: triglycerides.

## Data Availability

The original contributions presented in the study are included in the article, further inquiries can be directed to the corresponding author.

## Supplementary Materials

The following supporting information can be downloaded at: https://www.mdpi.com/article/10.3390/nu17010135/s1, Table S1: Comparison between the baseline values of the two groups. Table S2: Linear regression analysis results. Comparison between the final values and the intervention adjusting for the baseline values. Figure S1: Demographic questionnaire.
