# Supplementary material for: The Effect of Activity Tracking Apps on Physical Activity and Glycemic Control in People with Prediabetes Compared to Normoglycemic Individuals: A Pilot Study
Source: Nutrients. 2024 Dec 31;17(1):135. doi: 10.3390/nu17010135 (PMC11722914; doi:10.3390/nu17010135)
Supplement: Supplementary file 1 [file nutrients-17-00135-s001.zip › nutrients-3371680-supplementary.pdf]

Table S1. Comparison between the baseline values of the 2 groups.

	Normoglycemics Baseline (t0)	Prediabetics Baseline (t0)	*p*-Value
**Steps/day**, mean (SD)	6292.47 (3245.79)	6360.33 (2782.38)	0.95
**BMI (kg/m²)**, mean (SD)	31.10 (27.20; 33.40)	28.30 (25.05; 30.95)	0.09
**Visceral fat (kg)**, mean (SD)	10.42 (3.92)	9.83 (3.55)	0.64
**FPG (mg/dL)**, mean (SD)	86.26 (10.22)	108.50 (6.49)	**<0.001**
**Insulin (mUI/mL)**, median (25–75 percentile)	6.50 (3.50; 8.90)	10.05 (6.60; 11.90)	0.01
**HbA1c (%)**, mean (SD)	5.28 (0.32)	5.92 (0.24)	**<0.001**
**Cre (mg/dL)**, mean (SD)	0.86 (0.14)	0.89 (0.14)	0.52
**TCHOL (mg/dL)**, median (25–75 percentile)	204.00 (154.00; 223.00)	156.00 (142.75; 230.25)	0.36
**TRG (mg/dL)**, median (25–75 percentile)	90.00 (69.00; 114.00)	93.50 (74.00; 130.50)	0.40
**HDL-C (mg/dL)**, median (25–75 percentile)	58.00 (16.04)	55.00 (14.99)	0.56
**LDL-C (mg/dL)**, median (25–75 percentile)	122.00 (90.00; 142.00)	88.50 (73.50; 134.75)	0.14
**APOA1 (mg/dL)**, mean (SD)	156.68 (24.71)	152.39 (30.32)	0.64
**APOB (mg/dL)**, median (25–75 percentile)	80.70 (76.30; 93.30)	73.15 (64.03; 89.10)	0.13
**Lpa (mg/dL)**, median (25–75 percentile)	6.10 (2.70; 28.70)	9.25 (3.95; 40.13)	0.32
**ACR (mg/g)**, mean (SD)	6.15 (2.32)	10.39 (12.04)	0.14

ACR: Albumin/Creatinine ratio, APOA1: Apolipoprotein A1, APOB: Apolipoprotein B, BMI: Body Mass Index, Cre: Creatinine, FPG: Fasting Plasma Glucose, HbA1c: Glycated hemoglobin, HDL-C: High-density lipoprotein cholesterol, LDL-C: Low-density lipoprotein cholesterol, Lpa: Lipoprotein (a), TCHOL: Total Cholesterol, TRG: Triglycerides.

Table S2. Linear regression analysis results. Comparison between the final values and the intervention adjusting for the baseline values.

	Effect (Normoglycemics vs. Prediabetics)	95% CI	*p*-Value
**Steps (n/day)**	399.63	−588.98; 1388.24	0.417
**BMI (kg/m²)**	−0.199	−0.684; 0.285	0.409
**Visceral fat (kg)**	−0.217	−0.737; 0.303	0.403
**FPG (mg/dL)**	3.818	−4.440; 12.078	0.354
**Insulin (mUI/mL)**	0.214	−1.721; 2.150	0.823
**HbA1c (%)**	0 .181	0 .005; 0.356	**0.044**
**Cre (mg/dL)**	−0.004	−0.042; 0.033	0.828
**TCHOL (mg/dL)**	12.137	−4.296; 28.570	0.143
**TRG (mg/dL)**	−3.495	−23.474; 16.484	0.724
**HDL-C (mg/dL)**	3.344	−0.401; 7.089	0.078
**LDL-C (mg/dL)**	11.114	−3.165; 25.395	0.123
**APOA1 (mg/dL)**	1.941	−4.994; 8.877	0.573
**APOB (mg/dL)**	2.649	−4.114; 9.412	0.432
**Lpa (mg/dL)**	1.540	−1.755; 4.836	0.349
**ACR (mg/g)**	0 .249	−1.568; 2.067	0.782

Figure S1. Demographic questionnaire.

DEMOGRAPHIC QUESTIONNAIRE

1. **Full Name:** ___________________________
2. **Contact Phone:** ________________________

3. **Gender:** Male ☐
   Female ☐

4. **Age:** _______ years

5. **Weight:** _______ kg

6. **Height:** _______ cm

7. **What is your health status:**
   - Normoglycemic (FPG < 100 mg/dl, HbA1c <5.7%) ☐
   - Prediabetic (FPG 100-125 mg/dl, HbA1c 5.7-6.4%) ☐

8. **Please indicate if you have any previous medical history** (Hypertension, dyslipidemia, stroke, diabetes-myocardial infarction, etc.)
   ____
   ____
   ____
   ____

9. **Are you currently on any medication? If yes, please provide details of your medication.**
   ____
   ____
   ____
   ____
   ____
   ____
   ____
   ____
   ____

10. **How often do you exercise? (Includes walking at a fast pace >5km/h)**
    - Never ☐
    - Once a week ☐

- Twice a week ☐
- Three times a week ☐
- Four times a week ☐
- Five times a week ☐
- Six times a week ☐
- Seven times a week ☐

11. How many minutes do you spend exercising each time?

____

12. What type of exercise do you do?

____

13. Do you currently use or have you used in the past any activity tracking app?

- Yes ☐
- No ☐
